# Low-Dimensional Network Formation in Molten Sodium Carbonate

**DOI:** 10.1038/srep24415

**Published:** 2016-04-15

**Authors:** Martin C. Wilding, Mark Wilson, Oliver L. G. Alderman, Chris Benmore, J. K. R. Weber, John B. Parise, Anthony Tamalonis, Lawrie Skinner

**Affiliations:** 1 Department of Physics, University of Bath, Claverton Down, Bath BA2 7AY, UK; 2 Department of Chemistry, Physical and Theoretical Chemistry Laboratory, University of Oxford, South Parks Road, Oxford OX1 3QZ, UK; 3 X-ray Science Division, Argonne National Laboratory, Argonne IL60439, USA; 4 Materials Development, Inc., Arlington Heights IL 60004, USA; 5 Geosciences Department and Department of Chemistry, Stony Brook University, NY 11794-2100, USA

## Abstract

Molten carbonates are highly inviscid liquids characterized by low melting points and high solubility of rare earth elements and volatile molecules. An understanding of the structure and related properties of these intriguing liquids has been limited to date. We report the results of a study of molten sodium carbonate (Na_2_CO_3_) which combines high energy X-ray diffraction, containerless techniques and computer simulation to provide insight into the liquid structure. Total structure factors (*F*^*x*^(*Q*)) are collected on the laser-heated carbonate spheres suspended in flowing gases of varying composition in an aerodynamic levitation furnace. The respective partial structure factor contributions to *F*^*x*^(*Q*) are obtained by performing molecular dynamics simulations treating the carbonate anions as flexible entities. The carbonate liquid structure is found to be heavily temperature-dependent. At low temperatures a low-dimensional carbonate chain network forms, at *T* = 1100 K for example ~55% of the C atoms form part of a chain. The mean chain lengths decrease as temperature is increased and as the chains become shorter the rotation of the carbonate anions becomes more rapid enhancing the diffusion of Na^+^ ions.

Carbonatites are rarely occurring igneous liquids whose formation is dominated by molten carbonates derived from the Earth’s mantle. Although their occurrence is currently restricted to a single active volcano, Ol Doinyo Lengai in Tanzania, it is believed that this type of volcanism has occurred throughout geological history^1^,^2^,^3^,^4^. As recently observed eruptions confirm, these carbonatite liquids have low viscosity and low eruption temperatures and are believed to play a significant role in the geological evolution of other terrestrial planets^5^. The low viscosity, the high solubility of key elements such as P and the light rare earth elements and ability to dissolve volatile elements makes carbonatite liquids important agents for geochemical enrichment in the Earth’s mantle and they are closely linked to kimberlite genesis and diamond formation^6^,^7^. Economically, carbonatites are important as sources of rare metals including niobium, tantalum and uranium^2^,^3^ while molten carbonates are important in development of molten carbonate fuel cells and as battery electrolytes^8^. Despite their recognised importance the structure of these liquids is not well-known. The traditional view is that they are similar to molten salts with carbonate groups 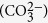 acting as anions and combining with metal cations^9^,^10^,^11^. This contrasts strongly with the silicate liquids which form the majority of terrestrial igneous liquids and are considered to be dominated by polymerised Si_*n*_O_*m*_ networks^12^.

An early X-ray diffraction (XRD) study provided estimates of the C-O, C-C and O-O distances for the carbonate anion and M-O, M-C and M-M distances in molten alkali (M^+^) carbonate liquids^13^. These X-ray data were interpreted in terms of the contact distances between the oxygen atoms in carbonate anions and the metal cations. The number of sites in contact with a single oxygen was found to increase systematically from Li^+^ to K^+^ and correlated with an increase in free volume allowing free rotation of anions. Vibrational spectroscopy performed on carbonate systems that form glasses hints at two populations of carbonate species, resembling those observed in crystalline configurations and those forming part of a network with the cations^14^,^15^, making their structures more complex than a typical molten salt. Simulations of carbonates which assume rigid anions with cations in interstices do not reflect the distortions suggested by spectroscopy^15^,^16^.

In this study we present the results of a series of state-of-the-art *in situ* XRD experiments on levitated carbonate liquids and similarly advanced computer simulations of the same liquids. The aim of this work is to address some key questions that have arisen from the previous studies. These include questions such as whether the liquids can be considered as simple molten salts, whether the internal 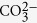 geometry directly affects both melt structure and/or dynamics, and also how the liquid structure contrasts with typical silicates^10^,^17^,^18^,^19^,^20^,^21^. The development of simulation models will allow, for example, temperature-dependent structural properties to be probed and will help steer future experimental investigation.

The structure of the sodium carbonate liquids is determined by high energy XRD (HEXRD) combined with a containerless levitation technique using four gas compositions. The *F*^*x*^(*Q*) obtained compares with the results obtained by Zarzycki^13^. Although the *Q*-range of this earlier study is limited, there is good agreement in the *position* of the first few peaks in the diffraction pattern. However the *intensities* of the peaks are substantially different and furthermore, the functions obtained here show peaks which are better resolved. The diffraction pattern shows well-resolved peaks at 1.57, 2.39, 3.44 and 6.14 Å^−1^ (Fig. 1(a)) and oscillations that persist to at least *Q* ~ 22 Å^−1^. The high *Q* oscillations are indicative of significant short-range ordering associated with the presence of stable molecular anions in the liquid, whilst the low *Q* features highlight the presence of ordering on longer length-scales.

The structure of the carbonate liquids can be usefully compared with silicates. Analogous sodium silicate glasses have been studied using both X-ray^21^ and neutron diffraction^17^,^18^,^22^. The first peak in *F*^*x*^(*Q*) for (Na_2_O)(SiO_2_) appears at around the same *Q* as the second peak in the corresponding carbonate^21^. This peak is dominated by Na-Na and Na-Si(C) contributions and, as a result, this strongly indicates that the Na-Na periodicites are equivalent in the two melts.

More insight into the liquid structure is obtained from the simulations of sodium carbonate liquids. Figure 1(a) shows *F*^*x*^(*Q*) generated from simulation at *T* = 1400 K compared with that from experiment (in supporting Ar gas). The simulated function shows the same “three peak” structure at *Q* ≤ 5Å^−1^ identified from experiment. Figure 1(a) also shows the contribution of the weighted partial structure factors. The peak at *Q* ~ 1.6 Å^−1^ is dominated by the O-C, O-O and O-Na contributions (although all six partials show intensity at this *Q*) and arises from the relatively long inter-ionic length-scale associated with the presence of the 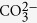 anions. The peak at *Q* ~ 3.7 Å^−1^ arises from a superposition of peaks in the O-Na and O-O partials. The strong oscillation in *F*^*x*^(*Q*) extending to high *Q* arises from a superposition of strong oscillations in both *S*_*OO*_(*Q*) and *S*_*CO*_(*Q*), the wavelengths of which correspond to the intramolecular O-O and C-O distances respectively.

The results of the simulation compare well with the experimental *F*^*x*^(*Q*) although there is a notable difference in the intensity of the peak at *Q* ~ 2.3 Å^−1^ which is dominated by C-Na and Na-Na contributions. Figure 1(b) shows how the simulated *F*^*x*^(*Q*) varies as a function of temperature. The peaks in *F*^*x*^(*Q*) at *Q*_1_ ~ 1.6 Å^−1^, *Q*_2_ ~ 2.3Å^−1^ and *Q*_3_ ~ 3.7 Å^−1^ show intensities which vary in different ways with temperature as shown in Fig. 1(c). The intensity of the peak at *Q*_2_ falls rapidly with temperature, whilst that of the peak at *Q*_3_ falls only weakly. Interestingly, the intensity of the peak at *Q*_1_ *increases* in intensity with temperature, a behaviour observed previously for so-called first sharp diffraction peaks^23^. The (small) differences in peak intensities between the experimental and simulation total scattering functions could, therefore, simply represent a slight shift in the melting point predicted by the present potential model away from the experimental value. Figure 1(d) shows the respective intensities of the weighted contributions from the six partial structure factors to the peak at *F*^*x*^(*Q*_1_) as a function of *T*. The key changes which contribute to *F*^*x*^(*Q*_1_) are in the O-O and Na-O partial functions. To understand the structural origin of this behaviour, however, we must move the analysis into real space.

Figure 2(a) shows the total radial distribution functions (rdf), *G*(*r*), generated in two ways; by combining the partial rdfs (*g*_*αβ*_(*r*) - shown in Fig. 2(b)) weighted by the number of electrons in each ion, and by Fourier transformation of *F*^*x*^(*Q*) (from Fig. 1b - mimicking the experimental procedure). The transformed functions are shown both with and without applying a Blackman windowing function. The windowing function acts to effectively remove the oscillations which arise from the artificial truncation of *F*^*x*^(*Q*), but results in significant peak broadening. Constructing *G*(*r*) *directly* from *g*_*αβ*_(*r*) removes any such truncation issues *but* is problematic in terms of applying the correct weighting functions. To highlight this effect the figure shows *G*(*r*) obtained using atomic (Na, C, O) and ionic (Na^+^, C^4+^, O^2−^) weightings respectively. The latter weighting generates functions similar with those obtained by Fourier transform. The peak at *r* ~ 1.3 Å is resolved entirely to *g*_*CO*_(*r*) (as would be expected for the intra-molecular C-O correlations). The peak at *r* ~ 2.4 Å is dominated by contributions from the O-O and Na-O rdfs. The Na-O rdf appears to have a relatively broad first peak consistent with a relatively distorted local environment for the sodium ions.

The transform of the experimental data compares well with the results of the simulation although the information that can be extracted from this real space data is limited. The C-O correlation at 1.3 Å is well-resolved, there is a low r shoulder to the peak at 2.3 Å. At higher *r* the partial contributions all overlap. Both *g*_*NaO*_(*r*) and *g*_*OO*_(*r*) contribute to the peak at *r* ~ 2.3 Å. The Na-O correlation is broader and, as a result, the change with temperature in intensity of the shoulder most likely reflects a change in the sodium environment. The mean Na-O coordination number from simulation varies from 5.82 at *T* = 1100 K to 4.96 at *T* = 1750 K compared with typical silicate values of 4.2^20^ and 3.6^18^ (from simulation) and 5.6^24^ from XAS studies.

Figure 2(c) shows the evolution with temperature of *g*_*CC*_(*r*). The most dramatic change occurs at *r* ~ 3.3Å with the clear peak at low *T* transforming to a weak shoulder at high *T*. The origin of this feature can be elucidated by considering molecular graphics “snapshots” taken from the simulation at different temperatures (see Fig. 3(b)). Bonds are drawn between the C atoms separated by *r* ≤ 4 Å (*i.e*. corresponding to the first peak in *g*_*CC*_(*r*), Fig. 2(b)). The carbonate anions are seen to form a low-dimensional network of (predominantly) chains. Figure 3(a) shows the distribution of the carbon chain lengths (shown as the number of carbon atoms in a chain of length *c*) at three temperatures with the inset to the figure showing the respective mean chain lengths as a function of *T*. As the temperature falls the number of longer chains increases (as does the mean chain length). Although these observations are primarily simulation results, they motivate further experimental (*e.g*. Raman spectroscopic, nuclear magnetic resonance) study in order to distinguish the different carbon environments.

To help understand how the observed network links with the system dynamics Fig. 4(a) shows the diffusion coefficients, *D*_*i*_, calculated from the respective mean-square displacements for the ionic species (*i* = O, C, Na) as a function of *T*^−1^. *D*_*C*_ and *D*_*O*_ are highly correlated, reflecting their formal association in the model whilst the Na^+^ ions show significantly higher diffusivities. The figure shows the Na^+^ diffusion coefficients obtained by Spedding and Mills^25^ via an open-ended capillary method. The raw data points are shown along with the original fit extended to match the present temperature range. The diffusivities obtained here are systematically lower than those obtained experimentally. To show the effect of the molecular nature of the anion, additional simulations are performed in which the harmonic springs which join the C-O and O-O atoms are increased (by a factor of 5), making the springs more stiff and, as a result, tending towards the rigid-molecule “limit” employed in previous models^16^. The effect of stiffening the intramolecular bonds is to significantly reduce 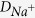 across the whole temperature range.

To further highlight the role of the 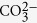 anion in the diffusion of the Na^+^ cations, we consider the *difference* in the respective O and C atom mean-squared displacements (see Fig. 4(b)), which contains information regarding the respective vibrational and rotational motions of the molecules^16^. As the temperature is increased the rotational/vibrational motion becomes more rapid and the long *t* limit is reached more rapidly. As the spring constants are again stiffened the motion becomes significantly slower, correlated with the slowing of the Na^+^ motion. As a result, the internal modes of the molecular anions appear crucial in facilitating the relatively rapid motion of the cations.

Figure 4(a) shows the comparison to Na^+^ diffusion coeffcients with those obtained from previous silicate simulations, (Na_2_O)(SiO_2_)_2_^20^,^26^ and (Na_2_O)(SiO_2_)_3_^20^ respectively. To promote a more direct comparison, the data from Horbach *et al*.^20^ is extrapolated into the temperature range studied here. In all three cases the observed silicate Na^+^ diffusion coefficients are orders of magnitude smaller than those observed in the carbonate suggesting diffusion is enhanced by the “paddle wheel” effects of asymmetric, rotating anions (see, for example^27^).

The combined simulation and HEXRD shows a liquid structure far more complicated than a simple ionic liquid with the 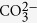 anions forming a low-dimensional network at low temperature. Although bridging has been suggested in carbonate liquids this has been assumed to link the anions and cations in the melt^15^. As shown by the changes in the *g*_*CC*_(*r*) the carbonate chains break up with increasing temperature allowing the anions to more freely rotate. The increased rotations “average out” the structure leading to greater O-O ordering on the length-scale corresponding to *Q* ~ 1.6 Å^−1^. As temperature is increased there is a fall in intensity of the peak at *Q* ~ 2.3 Å^−1^ which is associated with the increase in mobility of the Na^+^ ions as the carbonate network breaks up.

A variety of structural environments result from the flexibility of the carbonate anions and there is a strong temperature-dependence of structure (the formation of a low-dimensional carbonate network) which influences the diffusion. Furthermore, this diversity of structural environments that results could explain the high affinity for otherwise incompatible elements^14^. The temperature dependence of structure would indicate that these liquids are extremely *fragile*, with high temperature liquids dominated by Na^+^ diffusion enhanced by the rotation of asymmetric 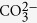 anions. Lower temperature liquids would be expected to show the increased influence of the carbonate chains and it will be interesting to investigate how their length may grow as the glass transition temperature is approached. Vitreous forms of carbonates would also be expected to be dominated by these carbonate networks.

## Methods

### Experimental Background

High energy X-rays (*E* > 100 *keV*) have been shown to be very effective in studying the structure of liquid and amorphous materials^28^. They reduce the corrections required to account for absorption and multiple scattering whilst their short wavelength provide scattering data to high values of the scattering vector, *Q*, (to *Q*_*max*_ > 20 Å^−1^) essential for good real-space resolution. Liquids have inherently low scattering and whilst high energy X-rays are an excellent tool for studying liquid structure the total scattering signal can be swamped by contributions from sample environments such as furnaces or sample containers. One, very productive approach is to combine high energy X-ray diffraction (HEXRD) with containerless techniques which eliminate the contribution of furnaces or similar sample environments. Combined HEXRD and containerless techniques enable studies of stable and metastable (supercooled) liquids to be undertaken.

In this study an aerodynamic levitation furnace is used, a 2–3 mm diameter bead of sample precursor is levitated in a divergent conical nozzle by a flowing levitation gas, the composition of which can be controlled. The sample is heated by a continuous wave 400 W CO_2_ laser and the temperature monitored by optical pyrometer operating at 0.85 *μ*m. The entire levitator is enclosed in a chamber and fully integrated into high energy beam lines at the APS (here 6-ID-D)^29^,^30^,^31^,^32^. The X-ray diffraction pattern of the liquid is measured in transmission mode with incident X-rays entering the chamber though a 1 cm aperture and emerging through a second window mounted opposite. Other apertures provide access to the levitation chamber for the pyrometer and for a video camera. The incident X-ray beam in these experiments had an energy of 100.099 keV, which corresponds to a wavelength of 0.1239 Å. Collimation was used to achieve an incident beam of 0.25 mm high and 0.5 mm wide. The entire 2D liquid diffraction pattern is detected on an vertically mounted Perkin Elmer XRD1621 Tl-doped CsI scintillator detector with 2048 × 2048 pixels of 200 *μ*m × 200 *μ*m set at a distance of 335.13 mm from the levitator nozzle. The detector distance, coordinates of the direct beam and the angle of tilt and rotation are refined by calibration using a crystalline CeO_2_ standard placed in the levitator nozzle.

**Figure 1:**
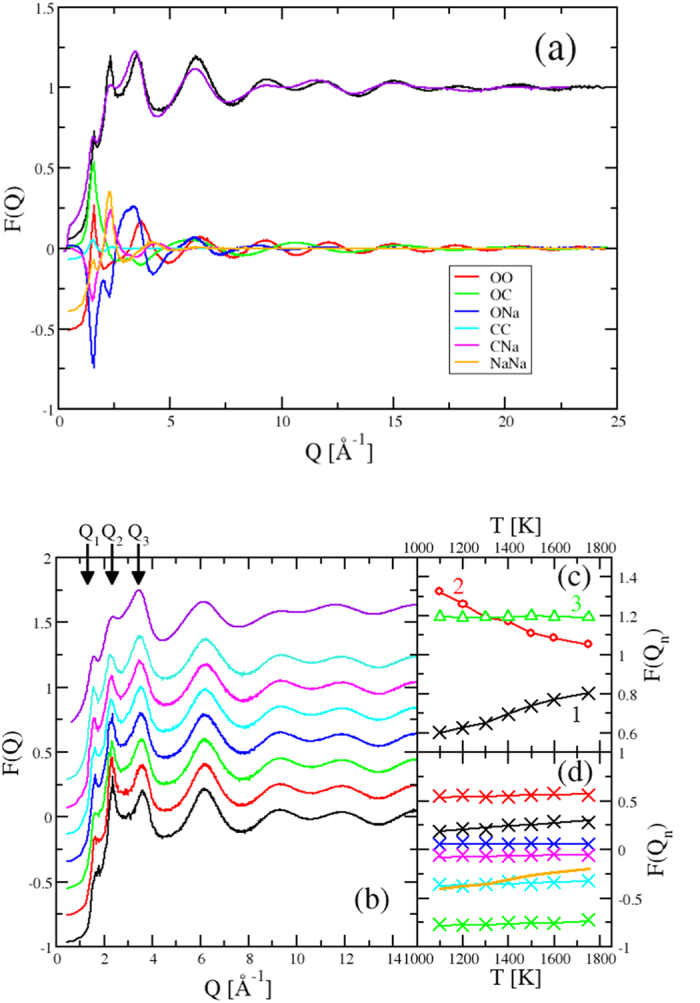
(**a**) The top two curves show the X-ray total structure factors from the present work obtained by experiment (purple line, levitated in Ar gas) and simulation (black line). Both of these curves are offset by one along the ordinate axis for clarity. The remaining curves show the weighted contributions of the six partial structure factors (identified in the legend) to the simulation total structure factor. (**b**) Temperature dependence of the X-ray total structure factor from simulation. The curves obtained at successive temperatures (from bottom to top, *T* = 1100 K, 1200 K, 1300 K, 1400 K, 1500 K, 1600 K, 175 K) are offset along the ordinate axis for clarity. The upper curve shows the experimental function obtained using Ar levitating gas. (**c**) The temperature evolution of the respective heights of the first three peaks in *F*^*x*^(*Q*) as indicated in the main panel as *Q*_1_, *Q*_2_ and *Q*_3_. (**d**) The temperature evolution of the intensities of the six partial structure factors contributing to the peak at *F*(*Q*_1_). Key: black - OO, red - CO, green - NaO, Blue - CC, cyan - NaC, magenta - NaNa, orange - total *F*(*Q*).

The structure factor has been obtained for liquid levitated using four different levitation gases; O_2_, CO_2_, Ar and a mixture of 80% CO_2_ and 20% CO. The CO_2_ and CO gases are 5% volume in argon. The temperatures, measured at the sample surfaces, were 1255 K for the pure and 80% CO_2_ gases, 1285 K for the pure O_2_, and 1328 K for the pure Ar. The 1D diffraction pattern were obtained by integrating over all the pixels in the 2D image using the Fit2D software package^33^ and *F*^*x*^(*Q*) then obtained using PDFgetX2 software^34^ and corrected by subtracting a background pattern collected with no sample in the nozzle but with flowing gas, and for Compton scattering. The Compton and form-factor contributions have a strong *Q*-dependence and are obtained from tabulated values^35^. The liquid densities, used to calculate the attenuation of the sample and to obtain the real space data by Fourier transform, were obtained from the studies of Liu and Lange^36^ and from Spedding^25^. Each diffraction measurement comprised a series of 60, 1 second frames with ideally five patterns collected and averaged for each sample. The levitation behavior of these very fluid liquids was different under different levitating gases. In pure O_2_ the drop oscillated considerably and tended to wet the levitator nozzle and, as a consequence, only two series were obtained for this gas composition. The most stable levitation was observed in pure Argon gas and under these conditions the drop neither oscillated nor stuck to the nozzle. Figure 1 shows very limited changes in structure are observed as a function of levitating gas. These small differences could result either from the nature of the levitating gas or from the (marginally) different levitation temperatures. The essential invariance of the scattering functions with levitating gas however indicates that there is no significant decomposition of the carbonate material in reducing conditions. The Na_2_CO_3_ used was Alfa Aesar 99.95–100.05%.

### Simulation Background

The potential model employed for the Na_2_CO_3_ simulation is based on that developed by Tissen and Janssen^16^ which uses a standard Born-Huggins-Mayer form with charges *Q*_*i*_ taken as *Q*_*Na*_ = 1.0e, *Q*_*C*_ = 1.54e and *Q*_*O*_ = −1.18e (giving 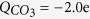 overall). In the original work^16^ the shape of the 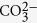 ion was imposed by using constraint dynamics to retain a fixed geometry. In the present work the basic trigonal planar geometry is imposed by employing harmonic springs which act between the C-O and O-O pairs in the molecular anion (as used previously in the study of sulphates^27^). The force constants controlling the respective C-O and O-O interactions may be varied. Simulations are performed over a temperature range *T* = 1100 K–1750 K at constant volume using the equation of state from Liu and Lange^36^.

*F*^*x*^(*Q*) is generated by combining the partial (Ashcroft-Langreth) structure factors (of which there are six for the three component system). These are calculated directly from the Fourier components of the ion densities, 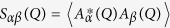, where 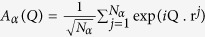. Total X-ray structure factors are constructed from weighted sums of these partial structure factors using X-ray form factors taken from standard sources^37^.

## Additional Information

**How to cite this article**: Wilding, M. C. *et al*. Low-Dimensional Network Formation in Molten Sodium Carbonate. *Sci. Rep*. **6**, 24415; doi: 10.1038/srep24415 (2016).

## Figures and Tables

**Figure 2:**
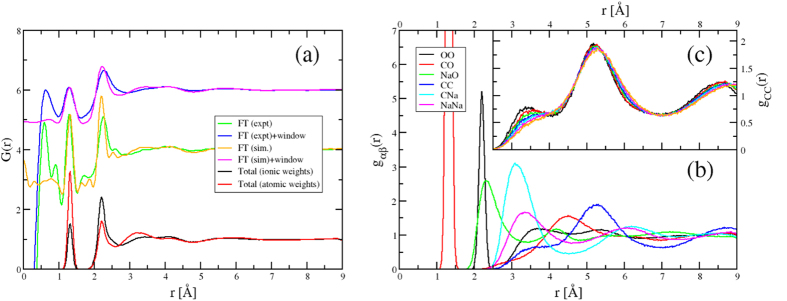
(**a**) Total pair distribution functions obtained from both experiment and simulation. The black and red curves are obtained directly from the partial pair distribution functions using ionic and atomic weightings respectively. The remaining curves are obtained by direct Fourier transformation of the experimental and simulated total scattering functions (with and without a window to force *F*(*Q*) → 0 at *Q*_*max*_). Successive curves are offset along the ordinate axis for clarity. (**b**) Partial pair distribution functions obtained from simulation at *T* = 1400 K. (**c**) The evolution of *g*_*CC*_(*r*) with *T* with the peak at *r* ~ 3.3 Å decreasing in intensity as the temperature is increased.

**Figure 3:**
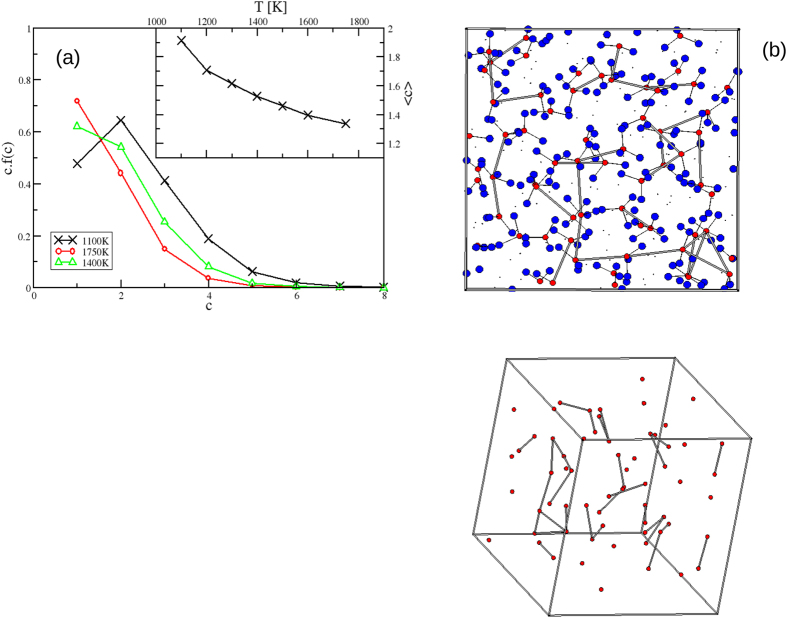
(**a**) The distribution of C-C… chain lengths, *c*, at three temperatures shown as *c.f*(*c*), the number of atoms in a chain of length *c*. The inset shows the mean chain length as a function of *T*. (**b**) Molecular graphics “snapshot” of a configuration taken from *T* = 1100 K. Key: red circles - C, blue circles - O, dots - Na. “Bonds” are drawn between C and O atoms from the same 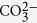 anion and between pairs of C atoms separated by *r* ≤ 4 Å (corresponding to the first peak in *g*_*CC*_(*r*) shown in Fig. 2(c)). The same configuration shown from an alternative angle and highlighting the carbon atoms only for clarity.

**Figure 4:**
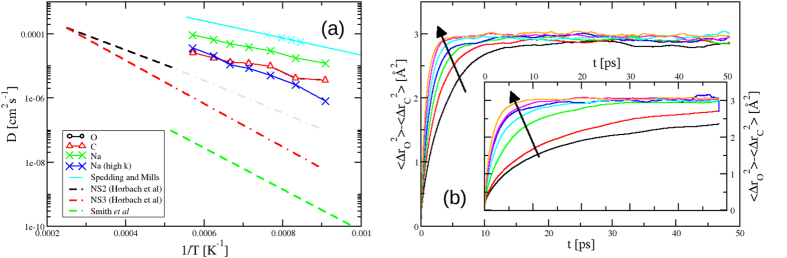
(**a**) Diffusion coefficients for the Na, C and O atoms (displayed as indicated in the legend) as a function of temperature compared with the experimental data for Na diffusion in Na_2_CO_3_ from Spedding and Mills^25^ (light blue; × - original data, line - original fit to the data extended to the present temperature range). The figure also shows the *D*_*Na*_ for a model with signficantly stiffer C-O and O-O intramolcular interation potentials. Comparison is also made to Na^+^ diffision on simulation studies of silicates performed by Horbach *et al*.^20^ and Smith *et al*.^26^, the former extrapolated to the temperature range studied here. (**b**) The difference in mean-squared displacements between the O and C atoms as a function of temperature (from *T* = 1100 K [black line] to *T* = 1750 K [orange line]). The inset shows the effect of stiffening the harmonic springs constraining the 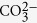 anions. The both panels the arrows highlight the changes in the respective function on *increasing* the temperature.
